# Patients over 65 years with Acute Complicated Calculous Biliary Disease are Treated Differently—Results and Insights from the ESTES Snapshot Audit

**DOI:** 10.1007/s00268-021-06052-0

**Published:** 2021-04-03

**Authors:** Gary A. Bass, Amy E. Gillis, Yang Cao, Shahin Mohseni, for the European Society for Trauma and Emergency Surgery (ESTES) Cohort Studies Group, Andreas Shamiyeh, Lena Rosetti, Günter Klimbacher, Bettina Klugsberger, Paul Healy, Conor Moriarty, Colm Power, Nauar Knightly, Arnold DK Hill, Desmond C Winter, Michael E Kelly, Ben E Creavin, Éanna J Ryan, Caoimhe C Duffy, Michael Sugrue, Michael Hugh Moore, Louise Flanagan, Jessica Ryan, Conor Keady, Brian Fahey, Kevin L McKevitt, Kevin Barry, Kevin C Conlon, Keno Mentor, Andrea Kazemi-Nava, Barbara Julies, Paul F Ridgway, Dara O Kavanagh, Mark Donnelly, Cathleen McCarrick, Umair Muhammad, Tara M Connelly, Paul C Neary, Sabina Magalina, Valerio Cozza, Antonio LaGreca, Daniele Gui, Alessia Malagnino, Mauro Zago, Mauro Montuori, Alan Biloslavo, Natasa Samardzic, Stefano Fracon, Davide Cosola, Nicolò de Manzini, Urânia Fernandes, Paulo Avelar, Rita Marques, Ana Sofia Esteves, André Marçal, Carina Gomes, Daniela Machado, Tobias Teles, Sofia Neves, Miguel Semiao, Rui Cunha, Jorge Pereira, Júlio Constantino, Milene Sá, Carlos Casimiro, Lidia Ionescu, Roxana Livadariu, Ludmila Stirbu, Radu Danila, Daniel Timofte, Bogdan Astefaniei, Aitor Landaluce Olavarria, Begoña Estraviz Mateos, Jaime Gonzalez Taranco, David Gomez, Jon Barrutia, Julio Zeballos, Dieter Morales Garcia, Ana Lozano Najera, Erik Gonzalez Tolaretxipi, Luis Tallon-Aguilar, José Pintor-Tortolero, Alejandro Sanchez-Arteaga, Virginia Duran-Muñóz Cruzado, Violeta Camacho-Marente, José Tinoco-Gonzalez, Anna Älverdal, Stefan Redeen, Shahin Mohseni, Ahmad Mohammad Ismail, Rebecka Ahl, Spyros Marinos, Naomi Warner, Rikhil Patel, Tania Magro, Romeshan Sunthareswaran, Andrei Mihailescu, Goran Pokusewski, Al exandru Leopold Bubuianu, Corneliu Dimitriu, Marius Paraoan, Arjun Desai, Katie Jones, Makhosini Mlotshwa, Kenny Ross, Simon Lambracos, Yegor Tryliskyy, Daniel C Cullinane

**Affiliations:** 1 Division of Traumatology, Emergency Surgery & Surgical Critical Care, Penn Presbyterian Medical Center, University of Pennsylvania, Philadelphia, PA 19104 USA; 2 School of Medical Sciences, Orebro University, 702 81 Orebro, Sweden; 3 Department of Surgery, Tallaght University Hospital, Dublin 24, Ireland; 4 Clinical Epidemiology and Biostatistics, School of Medical Sciences, Orebro University, 701 82 Orebro, Sweden; 5 Division of Trauma and Emergency Surgery, Department of Surgery, Orebro University Hospital, 701 85 Orebro, Sweden

## Abstract

**Background:**

Accrued comorbidities are perceived to increase operative risk. Surgeons may offer operative treatments less often to their older patients with acute complicated calculous biliary disease (ACCBD). We set out to capture ACCBD incidence in older patients across Europe and the currently used treatment algorithms.

**Methods:**

The European Society of Trauma and Emergency Surgery (ESTES) undertook a snapshot audit of patients undergoing emergency hospital admission for ACCBD between October 1 and 31 2018, comparing patients under and ≥ 65 years. Mortality, postoperative complications, time to operative intervention, post-acute disposition, and length of hospital stay (LOS) were compared between groups. Within the ≥ 65 cohort, comorbidity burden, mortality, LOS, and disposition outcomes were further compared between patients undergoing operative and non-operative management.

**Results:**

The median age of the 338 admitted patients was 67 years; 185 patients (54.7%) of these were the age of 65 or over. Significantly fewer patients ≥ 65 underwent surgical treatment (37.8% vs. 64.7%, *p* < 0.001). Surgical complications were more frequent in the ≥ 65 cohort than younger patients, and the mean postoperative LOS was significantly longer. Postoperative mortality was seen in 2.2% of patients ≥ 65 (vs. 0.7%, *p* = 0.253). However, operated elderly patients did not differ from non-operated in terms of comorbidity burden, mortality, LOS, or post-discharge rehabilitation need.

**Conclusions:**

Few elderly patients receive surgical treatment for ACCBD. Expectedly, postoperative morbidity, LOS, and the requirement for post-discharge rehabilitation are higher in the elderly than younger patients but do not differ from elderly patients managed non-operatively. With multidisciplinary perioperative optimization, elderly patients may be safely offered optimal treatment.

**Trial Registration:**

ClinicalTrials.gov (Trial # NCT03610308).

**Supplementary Information:**

The online version contains supplementary material available at 10.1007/s00268-021-06052-0.

## Introduction

The number of older people undergoing emergency noncardiac surgical procedures over the last 25 years has exceeded the rate of population aging. This increase is likely due to changing patient expectations, as well as advances in perioperative care. However, patients, age and pre-existing comorbidities remain significant predictors of adverse postoperative outcome [1–16].

In particular, elderly patients appear to accrue excess morbidity and mortality following gallbladder surgery [16]. Acknowledging acute complicated calculous biliary disease (ACCBD) as a common set of clinical problems presented to general surgeons, the Cohort Studies Group of the European Society for Trauma and Emergency Surgery (ESTES) set out to capture real-world data on the epidemiology, and contemporary management of these patients [17, 18]. Traditional medical school teaching identifies the 6Fs (fair, fat, fertile, female, forty, and family history) as phenotypic predictors of symptomatic gallstone disease [19]. Notably, however, the cohort presenting to European emergency departments with acute complicated biliary calculous conditions were older than this teaching suggests, with a median age of 67 years [17, 18].

Despite consensus in several guidelines, surgical practice patterns regarding older patients with ACCBD appear to vary between centers and even between individual surgeons [17, 18]. This prospective non-randomized observational cohort study aims to provide granular real-world data contrasting outcomes in patients over and under 65 years of age and comparing outcomes following operative and non-operative treatment strategies in patients over 65. Learnings from these data are presented as hypothesis-generating to stimulate further work investigating why definitive surgical therapy is not routinely offered to older patients and to create a context for practice management guidelines.

## Methods

### Study centers and patients

This study was an exploratory subgroup analysis from an international, multicenter, prospective non-randomized observational cohort study, conducted according to a previously published protocol (protocol registered at ClinicalTrials.gov, #NCT03610308). All participating centers provided local institutional review board approval or equivalent as a requirement of registration. Center and patient eligibility, as well as data capture, have been reported in previous publication [18].

The study included adult patients (over 18 years of age) presenting with acute complicated calculous biliary disease defined as one of the following diagnoses: American Association of Trauma (AAST) Severity Grade II or above acute calculous cholecystitis, choledocholithiasis, cholangitis, or biliary pancreatitis; subjected to any interventions ranging from conservative management to surgical or radiological interventions. The study excluded patients with uncomplicated biliary colic, biliary dyskinesia, or uncomplicated acute calculous cholecystitis (AAST Grade I).

### Statistical analysis

Descriptive and inferential statistical analyses were performed using Stata 15.1 (StataCorp LLC, College Station, TX, USA) and the jamovi project (www.jamovi.com, 2019) utilizing the R language for statistical computing. Effect estimates are presented as odds ratios (OR) with 95% confidence intervals and two-tailed *P* values. An alpha significance level of 0.05 was used throughout. Measures of central tendency were presented as mean [± standard deviation (SD); median, interquartile range (IQR)].

### Outcome measure

Outcomes were compared between patients aged under and ≥ 65 years and, within the ≥ 65 group, between patients who underwent operative or non-operative management. The primary outcome measure was index admission surgical treatment by cholecystectomy. Secondary outcome measures included the total LOS, the postoperative LOS (in whole days), postoperative and 30-day mortality rates, and a post-discharge rehabilitation requirement.

## Results

### Participating centers

Following an open call for participation by ESTES in May 2018, 38 centers expressed interest in participating. Of those, 25 centers completed the local ethics approval process and enrolled patients in the study. These centers came from 9 countries: Austria, Italy, Ireland, Romania, Spain, Sweden, Portugal, the United Kingdom, and the USA [18].

### Patient demographics and clinical characteristics

Three-hundred and thirty-eight individual patients admitted between October 1 and October 31 2018 were enrolled in the study and followed up until 120 days following admission.

Over half (54.7%) of the study cohort were age 65 years or older. The mean age in the ≥65 years group was 79 ± 8 years compared to 47 ± 12 years in the < 65 years group (*p* = 0.001). There was no statistically significant difference in sex between the groups (59.5% vs. 49.2%, *p* = 0.075). As depicted in Table 1, patients in the ≥ 65 years group had more comorbidities measured by their Charlson Comorbidity Index (CCI), were less fit for surgery based on their ASA score, were significantly more likely to have a history of ischemic heart disease, congestive heart failure, cerebrovascular disease, insulin-dependent diabetes mellitus, and chronic renal disease compared to patients under 65. Previous abdominal surgical history did not differ between patients under and ≥65 years (37% vs. 34%, *p* = 0.487) (Table 1).

**Table 1:** Patient Demographics, comparing those under and over 65 years

	< 65 years	≥ 65 years	Total	*p* value
Included patients* n* (%)	153 (45.3)	185 (54.7)	338 (100)	
Age, years, mean (SD)	47 (12)	79 (8.0)	65 (18)	< 0.001
Sex, Female* n* (%)	91 (59.5)	91 (49.2)	182 (53.8)	0.075
Charlson Comorbidity Index, median (IQR)	1.0 (0.0–2.0)	4.0 (4.0–6.0)	3.0 (1.0–5.0)	< 0.001
Age-adjusted Charlson Comorbidity Index, median (IQR)	3.0 (1.0–4.0)	9.0 (7.0–11.0)	6.0 (3.0–9.0)	< 0.001
*BMI (kg/m2), mean (SD)*	29.6 (7.2)	27.3 (6.2)	28.3 (6.8)	0.002
*ASA, n (%)*				< 0.001
1	53 (35.1)	9 (4.9)	62 (18.6)	
2	74 (49.0)	75 (41.2)	149 (44.7)	
3	18 (11.9)	77 (42.3)	95 (28.5)	
4	6 (4.0)	21 (11.5)	27 (8.1)	
*Diagnosis, n (%)*				0.065
Cholecystitis	68 (44.4)	86 (46.5)	154 (45.6)	
Gallstone pancreatitis	40 (26.1)	31 (16.8)	71 (21.0)	
CBD stone	25 (16.3)	36 (19.5)	61 (18.0)	
Cholangitis	16 (10.5)	31 (16.8)	47 (13.9)	
Mirizzi syndrome or bilioenteric fistula	4 (2.6)	1 (0.5)	5 (1.5)	
*AAST Cholecystitis Grade, n (%)*				0.048
1	0 (0.0%)	0 (0.0%)	0 (0.0%)	
2	58 (85.3%)	66 (76.7%)	124 (80.5%)	
3	4 (5.9%)	15 (17.4%)	19 (12.3%)	
4	6 (8.8%)	3 (3.5%)	9 (5.8%)	
5	0 (0.0%)	2 (2.3%)	2 (1.3%)	
Grade 3 or higher	10.0 (14.7%)	20.0 (23.3%)	30.0 (19.5%)	0.183^1^
*AAST Pancreatitis Grade, n (%)*				0.542
1	31 (81.6)	26 (83.9)	57 (82.6)	
2	6 (15.8)	4 (12.9)	10 (14.5)	
3	0 (0.0)	1 (3.2)	1 (1.4)	
4	1 (2.6)	0 (0.0)	1 (1.4)	

### Diagnosis

Cholecystitis was the most common (45.6%) diagnosis in the study cohort, followed by gallstone pancreatitis (21%), common bile duct stone (18%), and cholangitis (13.9%). Five patients (1.5%) had Mirizzi syndrome or bilioenteric fistula. There was no statistical difference in main admitting diagnosis between those under and ≥ 65 years (*p* = 0.065) (Table 1). The incidence of cholecystitis AAST grade III and V was higher in the ≥ 65 compared to the < 65 years group, 17.4% vs. 5.9% and 2.3% vs. 0% (*p* = 0.048), respectively. AAST grade IV cholecystitis was more common in the < 65 years group (8.8% vs. 3.5%, *p* = 0.048) (Table 1). There was no difference in the severity of pancreatitis between the groups based on the AAST grading (Table1).

### Surgical intervention

Of the 338 patients enrolled in the study, 50% underwent surgical intervention, while 50% had not received operative treatment by the end of the 120-day follow-up period. Patients younger than 65 were more likely to undergo index admission cholecystectomy than those ≥ 65 years (64.7% vs. 37.8%, *p* = 0.001). Cholecystectomy alone was performed in 99.3% of cases [18]. The use of laparoscopy did not differ between age groups (86.9% vs. 80.0%, *p* = 0.153). Conversion occurred in 14 (8.2%) cases, 5 in patients under 65 (5%) and 9 (12.9%) in those ≥ 65 years of age (*p* = 0.070). A further thirteen (7.6%) cholecystectomies were performed as open from the beginning of the procedure—eight in patients under 65 years and five in patients ≥ 65 (*p* = 0.909). Subtotal cholecystectomy was performed in 3 patients ≥ 65 and 1 in under 65 years (*p* = 0.180). It was not possible to ascertain the reasons for conversion, subtotal cholecystectomy, or primary open cholecystectomy. There was a trend towards shorter time to surgery from admission in the ≥ 65 group [days median (IQR): 1.0 (0.0–3.5) vs. 2.0 (1.0–6.2), *p* = 0.057] (Table 2).

**Table 2:** Surgical, endoscopic and radiologic intervention and outcomes, comparing those under and over 65 years

	< 65 years	≥ 65 years	Total	p value
Surgical Intervention, n (%)	99 (64.7)	70 (37.8)	169 (50.0)	< 0.001
*Surgical Approach, n (%)*				
Laparoscopic	86 (86.9)	56 (80.0)	142 (84.0)	0.153^1^
Laparoscopic converted to open	5 (5.0)	9 (12.9)	14 (8.2)	0.070^1^
Open	8 (8.1)	5 (7.1)	13 (7.6)	0.909^1^
*Type of surgery, n (%)*				
Cholecystectomy	98 (98.9)	67 (95.7)	165 (97.6)	0.399^1^
Subtotal cholecystectomy	1 (1.1)	3 (4.3)	5 (2.9)	0.180^1^
Admission to Surgery (days), median (IQR)	2.0 (1.0–6.2)	1.0 (0.0–3.5)	2.0 (0.0–5.5)	0.057
Endoscopy Intervention, n (%)	35 (22.9)	63 (34.1)	98 (29)	0.024
Admission to Endoscopy (days), median (IQR)	4.0 (2.0–5.0)	6.5 (2.2–11.0)	5.0 (2.0–8.0)	0.009
IR Intervention, n (%)	11 (7.2)	15 (8.1)	26 (7.7)	0.752
Admission to IR (days), median (IQR)	2.0 (0.0–6.0)	2.5 (1.2–9.2)	2.0 (0.5–7.0)	0.358
Postoperative LOS (days), median (IQR)	3.0 (2.0–6.0)	5.0 (3.8–9.2)	4.0 (3.0–7.0)	0.002
Total LOS (days), median (IQR)	7.0 (4.0–9.0)	7.5 (5.0–14.0)	7.0 (4.0–10.0)	0.039
*Disposition, n (%)*				
Home	149 (97.4)	157 (84.9)	306 (90.5)	< 0.001
Mortality	1 (0.7)	4 (2.2)	5 (1.5)	0.253
Convalescence	3 (2.0)	24 (13.0)	27 (8.0)	< 0.001

### Endoscopic management

Patients in the ≥ 65 group underwent endoscopic evaluation and management of the common bile duct more frequently than younger patients, (34.1% versus 22.9%, *p* = 0.024). Endoscopic Retrograde Cholangiopancreatography (ERCP) with duct clearance and sphincterotomy was the most commonly performed procedure (77.6%), followed by ERCP and stent placement (19.4%), and diagnostic EUS alone (3.1%). Of those patients undergoing ERCP, nine (9.2%) patients experienced complications, namely post-ERCP pancreatitis in six (6.1%) and bleeding in three (3.1%). No procedure was complicated by perforation. Time from admission to endoscopy was significantly longer in patients over the age of 65 years compared with patients under 65 [days Median (IQR): 6.5 (2.2–11.0) vs. 4 (2.0–5.0), *p* = 0.009] (Table 2).

### Interventional radiologic management

Interventional radiologic management of the gallbladder or common bile duct was undertaken in 26 (7.7%) of patients—11 patients under the age of 65 years and 15 patients ≥ 65 (*p* = 0.752). Cholecystostomy was performed in 23 (88.5%) of these cases, percutaneous radiologic drainage of a collection or abscess was performed in one (3.8%) patient, and percutaneous transhepatic cholangiography was performed in two (7.7%). No complication was recorded for patients undergoing interventional radiologic procedures. Time from admission to intervention radiology intervention did not differ between patients over the age of 65 years compared with patients under 65 [days median (IQR): 2.5 (1.2–9.2) vs. 2 (0.0–6.0), *p* = 0.358] (Table 2).

### Outcomes

Nineteen patients (5.6%) were admitted to ICU for organ failure during their hospital stay—10 (6.5%) patients under 65 and 9 (4.8%) ≥ 65 (*p* = 0.506). Both postoperative [days median (IQR): 5.0 (3.8–9.2) vs. 3.0 (2.0–6.0), *p* = 0.002] and total hospital length of stay [days median (IQR): 7.5 (5.0–14.0) vs. 7.0 (4.0–9.0), *p* = 0.039] (irrespective of diagnosis) was longer for patients ≥ 65 years (Table 2). Conversion to open cholecystectomy doubled the median (IQR) postoperative length of stay from 4.0 (2.0–6.0) to 8.0 (4.0–12.0) days (*p* = 0.006).

Five deaths were recorded (1.4%) in the total cohort– one (1.4%) patient in those suffering from gallstone pancreatitis, two (4.2%) in patients with cholangitis, and two (1.2%) patients with acute cholecystitis, both of which had an AAST Grade IV cholecystitis. One (0.58%) postoperative death occurred following cholecystectomy for AAST Grade IV cholecystitis. Four deaths occurred in patients ≥65 (2.2%), compared with one (0.7%) under 65 years (*p* = 0.253). Those patients who died had a significantly higher comorbidity burden, with a median (IQR) age-adjusted CCI (aaCCI) of 12.0 (11.0–12.0) versus 6.0 (3.0–8.0) in surviving patients (*p* < 0.001). Of the 333 patients surviving to discharge, significantly more patients ≥ 65 years had ongoing morbidity requiring post-acute convalescence or rehabilitation (13.0%), compared with 2% under the age of 65 (*p* < 0.001). Postoperative complications were reported more frequently in patients over 65 compared with younger patients without statistical significance (18.6% vs. 10.1%, *p* = 0.114) (Table 3).

**Table 3:** Operative and postoperative complications in patients under and over 65 years undergoing surgical intervention

	< 65 years	≥ 65 years	Total	*p* value
*Strasberg Classification of CBD Complication, n (%)*				0.183^1^
Cystic duct leak	2 (1.3)	5 (2.7)	7 (2.1)	
Lateral injury to CBD/CHD (Strasberg D)	1 (0.7)	0 (0.0)	1 (0.3)	
CBD/CHD division (Strasberg E1)	0 (0.0)	1 (0.5)	1 (0.3)	
*Postoperative, n (%)*				0.114^1^
No complication	89 (89.9%)	57 (81.4%)	146 (86.4%)	
Abscess	3 (3.0%)	4 (5.7%)	7 (4.1%)	
Bile duct complications	3 (3.0%)	6 (8.6%)	9 (5.3%)	
Hemorrhage	0 (0.0%)	1 (1.4%)	1 (0.6%)	
Wound infection	3 (3.0%)	2 (2.9%)	5 (3.0%)	
Enterotomy	1 (1.0%)	0 (0.0%)	1 (0.6%)	
Total	10 (10.1%)	13 (18.6%)	23 (13.6%)	

^1^Pearson’s Chi-squared test

## Demographics, management, and outcomes of the elderly subgroup

Patient demographic and clinical characteristics in patients over 65 years of age, dichotomized into surgical or non-operative management, are captured in Table 4. Patients who underwent cholecystectomy were younger (75 ± 7 vs. 81 ± 8 years, *p* < 0.001), male (64.3% vs. 42.6%, *p* = 0.004), and with fewer comorbidities [CCI median (IQR): 4.0 (3.0–5.0) vs. 5.0 (4.0–2.6), *p* = 0.031]. There was no difference in their fitness for surgery based on their preoperative ASA score assessment (*p* = 0.197) (Table 4). Acute cholecystitis was the most common diagnosis in the cholecystectomy group (74.3% vs. 29.6%, *p* < 0.001), while most non-operated patients presented with gallstone pancreatitis, common bile duct stone, or cholangitis (69.5% vs. 25.7%, *p* < 0.001). There was no difference in intervention radiology (*p* = 0.437) or endoscopy interventions (*p* = 0.813) between the cohorts (Table 5). No statistical differences in the total hospital length of stay or disposition after discharge was measured between the groups (Table 5).

**Table 4:** Demographics, treatment metrics and outcomes in patients over the age of 65 years, comparing those who underwent surgical intervention or not

	Not operated (*N* = 115)	Operated (*N* = 70)	Total (*N* = 185)	*p* value
Age, years, mean (SD)	81 (8)	75 (7)	79 (8.0)	< 0.001
Sex, Female* n* (%)	66 (57.4%)	25 (35.7%)	91 (49.2%)	0.004
Charlson Comorbidity Index, median (IQR)	5.0 (4.0–6.2)	4.0 (3.0–5.0)	4.0 (4.0–6.0)	0.031
Age-adjusted Charlson Comorbidity Index, median (IQR)	8.0 (7.0–10.0)	8.0 (6.0–9.0)	8.0 (7.0–10.0)	0.024
BMI (kg/m^2^), mean (SD)	27.0 (5.6)	28.6 (5.3)	27.6 (5.5)	0.049^1^
ASA,* n* (%)				0.197^2^
1	1 (3.1%)	4 (7.7%)	5 (6.0%)	
2	8 (25.0%)	23 (44.2%)	31 (36.9%)	
3	17 (53.1%)	19 (36.5%)	36 (42.9%)	
4	6 (18.8%)	6 (11.5%)	12 (14.3%)	
Diagnosis,* n* (%)				< 0.001^1^
Cholecystitis	34 (29.6%)	52 (74.3%)	86 (46.5%)	
Gallstone pancreatitis	27 (23.5%)	4 (5.7%)	31 (16.8%)	
Cholangitis	26 (22.6%)	5 (7.1%)	31 (16.8%)	
CBD stone	27 (23.5%)	9 (12.9%)	36 (19.5%)	
Mirizzi syndrome or bilioenteric fistula	1 (0.9%)	0 (0.0%)	1 (0.5%)	
AAST Cholecystitis Grade,* n* (%)				0.642^1^
1	0 (0%)	0 (0%)	0 (0%)	
2	26 (76.5%)	40 (76.9%)	66 (76.7%)	
3	7 (20.6%)	8 (15.4%)	15 (17.4%)	
4	1 (2.9%)	2 (3.8%)	3 (3.5%)	
5	0 (0.0%)	2 (3.8%)	2 (2.3%)	
Grade 3 or higher	8.0 (23.5%)	12.0 (23.1%)	20.0 (23.3%)	0.961^1^
AAST Pancreatitis Grade,* n* (%)				0.700^1^
1	23 (85.2%)	3 (75.0%)	26 (83.9%)	
2	3 (11.1%)	1 (25.0%)	4 (12.9%)	
3	1 (3.7%)	0 (0.0%)	1 (3.2%)	
4	0 (0%)	0 (0%)	0 (0%)	

^1^Linear Model ANOVA ^2^Trend test for ordinal variables

**Table 5:** Surgical, endoscopic and radiologic intervention and outcomes in patients over 65 years of age, comparing those who underwent surgical intervention or not

	Not operated (N = 115)	Operated (N = 70)	Total (N = 185)	*p* value
*Admission to IR (days)*				0.437^1^
IR, n (%)	9 (7.8%)	7 (10.0%)	18	
Mean (SD)	5.7 (11.5)	9.9 (9.2)	7.3 (10.6)	
*Admission to Endoscopy (days)*				0.813^1^
Endoscopy, n (%)	45 (39.1%)	19 (27%)	62	
Mean (SD)	9.0 (10.8)	9.8 (12.4)	9.3 (11.2)	
*Surgical Approach, n (%)*				
Laparoscopic		55 (78.6%)		
Laparoscopic converted to open		9 (12.9%)		
Open		6 (8.6%)		
*Type of surgery, n (%)*				
Cholecystectomy		66 (94.3%)		
Subtotal cholecystectomy		4 (5.7%)		
*Admission to ICU (days)*				
ICU admission, n (%)	1 (0.9%)	8 (11.4%)	9 (4.9%)	0.004
Median (IQR)	4.0 (4.0 to 4.0)	3.0 (1.8 to 8.0)	3 (2 to 8)	0.695^1^
*Total Length of Stay (days)*				0.538^1^
Mean (SD)	9.3 (7.6)	10.0 (9.3)	9.6 (8.3)	
Median (IQR)	7.0 (5.0 to 12.0)	7.0 (5.0 to 12.0)	7.0 (5.0 to 12.0)	
*Disposition, n (%)*				
Home	94 (81.7%)	63 (90.0%)	157 (84.9%)	0.128^1^
Mortality	3 (2.6%)	1 (1.4%)	4 (2.2%)	0.592^1^
Convalescence	18 (15.7%)	6 (8.6%)	24 (13.0%)	0.165^1^

^1^Linear Model ANOVA

## Discussion

There has been a disproportionate increase in the number of older adults within the world population over the last 50 years, with the global population aged 60 years or older projected to treble to nearly 2 billion people within the first half of this century [20]. In Europe alone, almost 30% of the population is predicted to be aged 65 or over by 2050 [20]. Geriatric patients frequently undergo emergency general surgery and accrue more postoperative complications and fatal outcomes than the general population [5, 13, 16]. It is thus highly relevant to develop and guide evidence-based patient-centered decision-making around emergency surgical care [21]. ACCBD is a relatively frequent diagnosis in patients over 65 years, and while some consensus guidance exists, age is not considered to be a contraindication to operative management [26].

As evidenced by a significantly higher CCI and age-adjusted CCI in the current study cohort, pre-existing cardiovascular, endocrine, and renal comorbidities are more frequent in older than younger patients [9–11]. While a direct correlation has yet to be studied between aaCCI and morbidity and mortality following emergency cholecystectomy, strong evidence exists supporting aaCCI as a robust predictor of poor outcomes following elective gynecologic, oncologic gastrointestinal, and emergency orthopedic surgery [22–25]. Indeed, the observed case fatality rate in our study was 2.2% in patients over 65 years, a postoperative complication occurred in 18.6%, and aaCCI was positively correlated with higher mortality and complication rates.

International guidelines, which advocate for index admission cholecystectomy, make no specific recommendations for older patients [2]. In the current cohort of patients admitted for acute complicated biliary calculus disease, index admission cholecystectomy was achieved only in 37.8% cases in patients 65 years of age and older. Subgroup analysis of the over 65 years cohort detected no difference in fitness for surgery by anesthesia assessment (as measured by ASA classification score) or difference in severe cholecystitis incidence (AAST Grade 3–5). However, the non-operative group was found to be older. This finding is of particular importance since other gallstone complications (e.g., gallstone pancreatitis, common bile duct stone with or without concomitant cholangitis) were managed by percutaneous cholecystostomy or ERCP alone. Several studies have shown better outcomes in those who received early cholecystectomy following duct clearance for gallstone pancreatitis, and the Tokyo Guidelines (2018) advise index admission cholecystectomy in this instance [26, 27]. A recent comparison of one-stage ERCP and cholecystectomy versus index admission ERCP with planned interval elective day-case cholecystectomy demonstrated a readmission rate over 20% in the latter group, due to complications relating to the retained gallbladder as a stone reservoir [28]. Regrettably, our current data did not report readmission rate, but given the overlap in patient populations, we would expect similar rates of disease recidivism in the non-operated cohort. However, there was no difference in postintervention complications between the cohorts. These are important factors to consider when deferring cholecystectomy in the elderly frail patient since another physiologic hit from recurrent disease could be devastating. Further, as a temporary measure of source control compared to cholecystectomy, percutaneous cholecystostomy has been shown to be associated with more complication and mortality in the elderly population [29]. Such interventions should be reserved for patients that fulfill the criteria for absolute contraindication for anesthesia or surgery [30].

Notably, recovery following acute illness and mainly surgical intervention may be prolonged in the elderly population, as a consequence of a loss of strength, mobility, and functional capacity [31–34]. However, there was no difference hospital length of stay, and post-discharge disposition comparing those patients over 65 years of age subjected to cholecystectomy versus their counterparts. Thus, it appears that underlying frailty, not surgical stress, might be the principal factor predicting recovery from acute illness.

The growing elderly patient population poses challenges to physicians and healthcare systems worldwide. While some risk factors, such as age and comorbidities, cannot be modified, other components of care such as multidisciplinary management in close collaboration with gerontology colleagues, time to the operating room and early physiologic optimization have been shown to improve outcomes. To that end, The American College of Surgeons has launched a geriatric verification program for hospitals and the American Geriatrics Society and the American College of Surgeons have issued joint guidelines on optimal care of the geriatric surgical patient based on analysis of retrospective administrative NSQIP data [35, 36]. Finally, with the linkage between higher hospital and surgeon volume of emergency surgical cases and improved outcome, especially in geriatrics, designated centers or indeed acute surgical service reorganization at a local level should be considered for this patient population in the future [37–39].

## Conclusion

Although this study is limited by its intention and design as a descriptive study, granular ‘real world’ data illustrate the challenges faced by elderly patients with AACBD. Excess morbidity, mortality, length of postoperative hospitalization, and a greater need for rehabilitation are seen in these patients. Guidelines considering age and comorbidities, routine use of prognostic tools and frailty indices in mitigating risk or pre-rehabilitation, and anticipating the requirement for enhanced supports in this patient cohort is warranted.

### Supplementary Information

Below is the link to the electronic supplementary material.

Supplementary file1 (DOCX 14 kb)

## Data Availability

The datasets used and analyzed during the current study are available from the corresponding author on reasonable request. This prospective, observational, multicenter audit was conducted in line with a pre-specified protocol which was registered with ClinicalTrials.gov (Trial # NCT03610308).

## Appendix 1

**Table:** Collaborating Group Membership

Country	Center name	Authors
Austria	Kepler University Clinic	Andreas Shamiyeh, Lena Rosetti, Günter Klimbacher, Bettina Klugsberger
Ireland	Beaumont Hospital Dublin	Paul Healy, Conor Moriarty, Colm Power, Nauar Knightly, Arnold DK Hill
Ireland	SVUH Institute for Clinical Outcomes Research and Education, Dublin	Desmond C Winter, Michael E Kelly, Ben E Creavin, Éanna J Ryan, Caoimhe C Duffy
Ireland	Letterkenny University Hospital	Michael Sugrue, Michael Hugh Moore, Louise Flanagan
Ireland	Mayo University Hospital, Castlebar, Mayo	Jessica Ryan, Conor Keady, Brian Fahey, Kevin L McKevitt, Kevin Barry
Ireland	Saint Vincent’s University Hospital, Dublin	Kevin C Conlon, Keno Mentor, Andrea Kazemi-Nava, Barbara Julies
Ireland	Tallaght University Hospital, Dublin	Paul F Ridgway, Dara O Kavanagh, Mark Donnelly, Cathleen McCarrick, Umair Muhammad, Tara M Connelly, Paul C Neary
Italy	Fondazione Policlinico Universitario "A. Gemelli" IRCCS	Sabina Magalina, Valerio Cozza, Antonio LaGreca, Daniele Gui
Italy	Policlinico San Pietro, Bergamo, Milano	Alessia Malagnino, Mauro Zago, Mauro Montuori
Italy	Clinica Chirurgica, Trieste University Hospital, Trieste, Italy	Alan Biloslavo, Natasa Samardzic, Stefano Fracon, Davide Cosola, Nicolò de Manzini
Portugal	Centro Hospitalar de Trás-os-montes e Alto Douro	Urânia Fernandes, Paulo Avelar, Rita Marques, Ana Sofia Esteves, André Marçal, Carina Gomes
Portugal	Centro Hospitalar Cova de Biera, Covilha	Daniela Machado, Tobias Teles, Sofia Neves, Miguel Semiao, Rui Cunha
Portugal	Centro Hospitalar Tondela Viseu	Jorge Pereira, Júlio Constantino, Milene Sá, Carlos Casimiro
Romania	St. Spiridon Emergency Universitary Hospital, Iasi	Lidia Ionescu, Roxana Livadariu, Ludmila Stirbu, Radu Danila, Daniel Timofte, Bogdan Astefaniei
Spain	Hospital Urduliz	Aitor Landaluce Olavarria, Begoña Estraviz Mateos, Jaime Gonzalez Taranco, David Gomez, Jon Barrutia, Julio Zeballos
Spain	University Hospital "Marques de Valdecilla"	Dieter Morales Garcia, Ana Lozano Najera, Erik Gonzalez Tolaretxipi
Spain	Hospital Virgen del Rocío	Luis Tallon-Aguilar, José Pintor-Tortolero, Alejandro Sanchez-Arteaga, Virginia Duran-Muñóz Cruzado, Violeta Camacho-Marente, José Tinoco-Gonzalez
Sweden	Jönköping Medical Center	Anna Älverdal
Sweden	Linköping University Hospital	Stefan Redeen
Sweden	Örebro University Hospital	Shahin Mohseni, Ahmad Mohammad Ismail, Rebecka Ahl
UK	Stoke Mandeville Hospital	Spyros Marinos, Naomi Warner, Rikhil Patel, Tania Magro, Romeshan Sunthareswaran
UK	Tameside General Hospital	Andrei Mihailescu, Goran Pokusewski, Alexandru Leopold Bubuianu
UK	Wrightington, Wigan and Leigh NHS Foundation Trust	Corneliu Dimitriu, Marius Paraoan
UK	Worthing Hospital	Arjun Desai, Katie Jones, Makhosini Mlotshwa, Kenny Ross, Simon Lambracos, Yegor Tryliskyy
USA	Marshfield Clinic	Daniel C. Cullinane

## Abbreviations

AAST: American Association for the Surgery of Trauma
aaCCI: Age-adjusted Charlson comorbidity index
ACCBD: Acute Complicated Calculous Biliary Disease
CHSA: Canadian Study of Health and Aging Frailty Index
ERCP: Endoscopic Retrograde Cholangiopancreatography
ESTES: European Society of Trauma and Emergency Surgery
ICU: Intensive Care Unit
IQR: Interquartile range
p-POSSUM: Portsmouth Physiological and Operative Severity Score for the Enumeration of Mortality and Morbidity
