# Supplementary material for: Patients over 65 years with Acute Complicated Calculous Biliary Disease are Treated Differently—Results and Insights from the ESTES Snapshot Audit
Source: World J Surg. 2021 Apr 3;45(7):2046–55. doi: 10.1007/s00268-021-06052-0 (PMC8154793; doi:10.1007/s00268-021-06052-0)
Supplement: Supplementary file 1 — Supplementary file1 (DOCX 14 kb) [file 268_2021_6052_MOESM1_ESM.docx]

| Authors |
| --- |
| Andreas Shamiyeh, Lena Rosetti, Günter Klimbacher, Bettina Klugsberger |
| Paul Healy, Conor Moriarty, Colm Power, Nauar Knightly, Arnold DK Hill |
| Desmond C Winter, Michael E Kelly, Ben E Creavin, Éanna J Ryan, Caoimhe C Duffy |
| Michael Sugrue, Michael Hugh Moore, Louise Flanagan |
| Jessica Ryan, Conor Keady, Brian Fahey, Kevin L McKevitt, Kevin Barry |
| Kevin C Conlon, Keno Mentor, Andrea Kazemi-Nava, Barbara Julies |
| Paul F Ridgway, Dara O Kavanagh, Mark Donnelly, Cathleen McCarrick, Umair Muhammad, Tara M Connelly, Paul C Neary |
| Sabina Magalina, Valerio Cozza, Antonio LaGreca, Daniele Gui |
| Alessia Malagnino, Mauro Zago, Mauro Montuori |
| Alan Biloslavo, Natasa Samardzic, Stefano Fracon, Davide Cosola, Nicolò de Manzini |
| Urânia Fernandes, Paulo Avelar, Rita Marques, Ana Sofia Esteves, André Marçal, Carina Gomes |
| Daniela Machado, Tobias Teles, Sofia Neves, Miguel Semiao, Rui Cunha |
| Jorge Pereira, Júlio Constantino, Milene Sá, Carlos Casimiro |
| Lidia Ionescu, Roxana Livadariu, Ludmila Stirbu, Radu Danila, Daniel Timofte, Bogdan Astefaniei |
| Aitor Landaluce Olavarria, Begoña Estraviz Mateos, Jaime Gonzalez Taranco, David Gomez, Jon Barrutia, Julio Zeballos |
| Dieter Morales Garcia, Ana Lozano Najera, Erik Gonzalez Tolaretxipi |
| Luis Tallon-Aguilar, José Pintor-Tortolero, Alejandro Sanchez-Arteaga, Virginia Duran-Muñóz Cruzado, Violeta Camacho-Marente, José Tinoco-Gonzalez |
| Anna Älverdal |
| Stefan Redeen |
| Shahin Mohseni, Ahmad Mohammad Ismail, Rebecka Ahl |
| Spyros Marinos, Naomi Warner, Rikhil Patel, Tania Magro, Romeshan Sunthareswaran |
| Andrei Mihailescu, Goran Pokusewski, Alexandru Leopold Bubuianu |
| Corneliu Dimitriu, Marius Paraoan |
| Arjun Desai, Katie Jones, Makhosini Mlotshwa, Kenny Ross, Simon Lambracos, Yegor Tryliskyy |
| Daniel C. Cullinane |
